# Retention of Urine in the Fœtus

**Published:** 1866-02

**Authors:** 


Retention of Urine in the Fœtus.—M. Depaul related to the Biological Society a case in which a woman was delivered of an eight months' child presenting a great enlargement of the abdomen; there was a very little amniotic fluid. The child died soon after being born. The bladder was about 4½ inches long and 2¾ wide, and was full of urine. The ureters were also irregularly enlarged, resembling at first sight the intestinal convolutions, and contained urine. The kidneys, especially the left, were also much distended; they were transformed into cysts with thin walls, filled also with urine. The cause of this distension was found to be an imperforate state of the urethra at the junction of the muscular and prostatic portions. More than a pint of urine was removed.—*Gaz. Med. de Paris, July* 15, 1865.

